# Akute und chronische kardiale Beteiligung bei COVID-19

**DOI:** 10.1007/s00117-021-00913-4

**Published:** 2021-09-16

**Authors:** Dietrich Beitzke

**Affiliations:** Universitätsklinik für Radiologie und Nuklearmedizin, Medizinische Universität Wien, Währinger Gürtel 18–20, 1090 Wien, Österreich

**Keywords:** Kardiovaskuläre Erkrankung, Myokarditis, Perikarditis, Kardiale Magnetresonanztomographie, Kardiale Computertomographie, Cardiovascular disease, Myocarditis, Pericarditis, Cardiac magnetic resonance imaging, Cardiac computed tomography

## Abstract

**Hintergrund:**

Neben pulmonalen Manifestationen ist eine COVID-19-Infektion (Coronavirus-Krankheit 2019) häufig mit kardiovaskulären Komplikationen bzw. einer kardiovaskulären Beteiligung assoziiert. Das Herz kann im Rahmen einer Infektion sowohl direkt im Rahmen einer Myokarditis oder Perikarditis und auch im Rahmen von Hypoxie, Fieber, Volumenbelastungen oder thrombembolischer Komplikationen involviert werden. Bestehende kardiovaskuläre Grunderkrankungen haben zudem einen maßgeblichen Einfluss auf die Prognose von COVID-19-infizierten Patienten.

**Methode:**

Diese Übersichtsarbeit basiert auf einer umfassenden Literaturrecherche in der PubMed-Datenbank zu kardialen Beteiligungen und kardialen Komplikationen einer COVID-19-Infektion sowie deren Abgleich mit eigenen Erfahrungen.

**Ergebnisse und Schlussfolgerung:**

Je nach Schweregrad der Infektion werden kardiale Beteiligungen im Rahmen einer COVID-19-Infektion mit bis zu 50 % durchaus häufig beobachtet. Neben der Echokardiographie als Untersuchungsmethode der ersten Wahl stellen die kardiale Magnetresonanztomographie (MRT) zur Beurteilung der myokardialen Struktur und die kardiale Computertomographie (CT) zur Beurteilung der Koronararterien bzw. zum Ausschluss eines intrakardialen Thrombus bedeutende Untersuchungsmodalitäten dar. Die wichtigsten kardialen Manifestationen einer COVID-19-Infektion sind entzündliche und ischämische Pathologien. Deren bildgebende Diagnostik spielt sowohl im akuten als auch im postinfektiösen Stadium eine bedeutende Rolle.

## Abstract (en)

**Background:**

Infections with coronavirus disease 2019 (COVID-19) most frequently affect the lungs but may also result in cardiovascular involvement and cardiovascular complications. The heart can either be involved as part of a systemic infection or directly involved due to myocarditis or pericarditis as well as in hypoxia, volume overload, fever or thromboembolic complications. Moreover, pre-existing underlying cardiovascular diseases have a substantial influence on the prognosis of patients with COVID-19 infections.

**Method:**

This review article is based on a comprehensive literature search in the PubMed database on cardiac involvement and cardiac complications of COVID 19 infections, enriched by experiences in dealing with this disorder.

**Results and conclusion:**

Depending on the severity of the infection, cardiac involvement in a COVID 19 infection is observed in up to 50% of the patients. Besides echocardiography as the first-line examination method, cardiac magnetic resonance imaging (MRI) for assessment of the myocardial structure and cardiac computed tomography (CT) for assessment of coronary arteries and to rule out intracardiac thrombus formation represent important imaging modalities. The most important cardiac manifestations in COVID 19 infections are ischemic and inflammatory diseases. The imaging diagnostics play an important role in the acute as well as in the postinfectious phases.

SARS-CoV-2-Infektionen sind neben der Lungenerkrankung häufig mit kardiovaskulären Komplikationen assoziiert. Diese basieren im akuten Stadium hauptsächlich auf einer stark gesteigerten Zytokinfreisetzung, welche mit einer vaskulären Inflammation und einer erhöhten Neigung zu Thromben und damit auch thrombembolischen Ereignissen einhergeht [1]. Neben der bekannten Neigung zu SARS-COV-2-assoziierten Thromboembolien in der pulmonalen und der arteriellen Strombahn geht dies auch mit kardiovaskulären Ereignissen einher. Daneben führt die Virusausbreitung zu einer generalisierten vaskulären Entzündungsreaktion. Bei den betroffenen Patienten können somit sowohl ischämische Zustandsbilder (aggraviertes akutes Koronarsyndrom oder Myokardinfarkte ohne Koronarobstruktion [MINOCA]) als auch nichtischämische Pathologien wie Myokarditis oder Perikarditis auftreten [2]. Auch bereits bestehende kardiovaskuläre Vorerkrankungen stellen einen hochrelevanten Risikofaktor für ein verschlechtertes Outcome einer SARS-CoV-2-Infektion dar.

Neben der Computertomographie (CT) der Lunge und der Pulmonalgefäße sind die CT und auch die Magnetresonanztomographie (MRT) des Herzens wichtige Untersuchungsmodalitäten bei Patienten mit bestehender oder überstandener COVID-19 (Coronavirus-Krankheit 2019; [2]). Der folgende Artikel gibt einen kurzen Überblick über die kardialen Komplikationen bei Patienten mit COVID-19 bzw. nach durchgemachter COVID-19-Infektion und anhaltenden Symptomen (Long-COVID).

## Pathophysiologie einer SARS-CoV-2-Infektion in Bezug auf das Herz

Das kardiovaskuläre System wird bei einer SARS-CoV-2-Infektion in vielfältiger Weise mit einbezogen und geschädigt. Zum einen spielt hier eine direkte Virustoxizität eine Rolle, zum anderen eine Beteiligung des Herzens im Rahmen des Zytokinsturms, mit dem die Infektion assoziiert ist. Bei diesem wiederum spielen Hypoxie durch (mikro)vaskuläre Schädigung bzw. Schäden durch hohes Fieber, Volumenbelastung und Medikamentenwechselwirkungen eine wesentliche Rolle. Insbesondere eine Rechtsherzbelastung durch pulmonale Infiltrate und Fieber kann das Outcome bei vorgeschädigten Herz negativ beeinflussen, besonders in der akuten Phase [3].

## Kardiale Bildgebung im akuten Stadium

Kardiale Bildgebung bei SARS-CoV-2-Patienten ist im akuten Stadium der Erkrankung meistens bei Anstieg des Troponin‑T im Serum bzw. klinische Symptome wie Thoraxschmerz oder kardiale Dekompensation indiziert, ebenso bei neu aufgetretenen Arrhythmien.

Der pulmonale initiale Eintritt des Virus wird durch pulmonale ACE2-Rezeptoren vermittelt. Diese werden auch im Herz und den Blutgefäßen exprimiert und könnten somit eine Beteiligung durch SARS-CoV‑2 erklären [4]. Eine myokardiale Beteiligung kann auch über ischämische und Nicht-Ischämie-Kaskaden ausgelöst werden. Bei Letzteren spielen der Zytokinsturm und eine direkte virusvermittelte Myokarditis eine Rolle [4].

### Akute Myokarditis bei COVID-19

Eine akute inflammatorische, myokardiale Beteiligung im Rahmen SARS-CoV-2-Infektion, gemessen an einer kardialen Enzymauslenkung, wird bei bis zu 12 % der Patienten beschrieben [5]. Dieser Anteil steigt bei intensivpflichtigen Patienten [5]. Auch in Autopsiestudien konnte bei diesen Patienten das Virusgenom im Myokard nachgewiesen werden [6].

**Figure 1:**
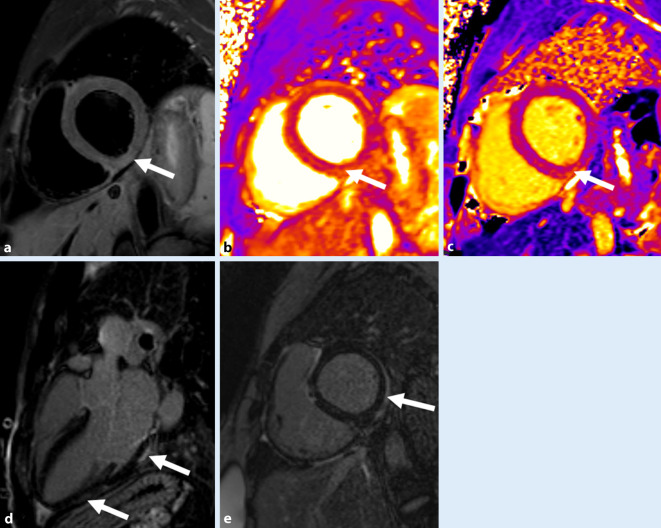

Die Bildgebung der akuten Myokarditis im Rahmen von SARS-CoV‑2 unterscheidet sich nicht von der bei *gewöhnlicher* Myokarditis und ist dementsprechend MR-basiert (Abb. 1). Der Nachweis einer akuten Entzündung erfolgt über die modifizierten Lake-Louise-Kriterien und benötigt im akuten Stadium den Nachweis eines T1- und eines T2-Kriteriums, um jeweils Ödem und Nekrose nachzuweisen [7]. Die Durchführung der kardialen MRT unterliegt natürlich, falls eine Infektion vorliegt, streng den lokalen Hygienevorschriften und muss mit Blick auf die weitere Behandlung des Patienten für klinische Entscheidungen erforderlich sein [8].

In ersten Studien während der Pandemie konnte gezeigt werden, dass sich die Verteilung der Myokardnekrosen bzw. die Verteilung der postmyokarditischen Narben geringgradig von der gewöhnlichen viral bedingten Myokarditis unterscheidet. So wurden in ersten Publikation über SARS-CoV-2-Infektionen mit kardialer Beteiligung Narbenbildungen vorwiegend an der Hinterwand des linken Ventrikels nachgewiesen [9]. Die Häufigkeit pathologischer Veränderungen in der Herz-MRT nach einer durchgemachten COVID-19-Infektion wird in der größten derzeit vorliegenden, prospektiven Serie an insgesamt 100 Patienten mit 60 % angegeben [10]. Hierbei zeigen sich einerseits milde Ventrikeldilatationen, andererseits reduzierte EF-Werte. Strukturelle Abnormitäten, wie erhöhte T1-Relaxationswerte in Mapping-Sequenzen bzw. der Nachweis von „late gadolinium enhancement“ (LGE) konnte in 73 % (T1) bzw. 32 % (LGE) gezeigt werden (Abb. 1). Zudem fanden sich kurz nach der Infektion noch Hinweise auf ein zumindest geringes Myokardödem (erhöhte T2-Relaxationswerte in den Mapping-Sequenzen) in 60 % der Patienten.

In der bisher größten Analyse von insgesamt 148 hospitalisierten Patienten die im Verlauf ihrer symptomatischen SARS-CoV-2-Infektion eine Troponin-Auslenkung im Labor zeigten, konnte in der kardialen MRT in etwa 50 % keine strukturelle Pathologie festgestellt werden [11]. Mittels LGE wurden in den restlichen 50 % verschiedene Muster der myokardialen Schädigung nachgewiesen werden. Hierbei wiederum zeigte der größte Teil der Patienten eine postinflammatorische Narbenverteilung [11]. Des Weiteren wurden postischämische oder unspezifische Narbenbildungen detektiert. Zudem wiesen Troponin-T-positive Patienten mit COVID-19 einen größeren rechten Ventrikel auf als eine nicht Troponin-T-positive COVID-19-positive Kontrollkohorte.

### Differenzialdiagnosen der akuten Myokarditis bei COVID-19

Neben dem postinflammatorischen Narbenmuster können Troponin-T-positive Patienten nach SARS-CoV-2-Infektion auch ein postischämisches Narbenmuster zeigen. Die Pathophysiologie der Ischämie während einer SARS-CoV-2-Infektion wird durch einen prothrombotischen Zustand getriggert, welcher in Kombination mit Hypoxie und Fieber im Rahmen der Infektion einen Myokardinfarkt hervorrufen kann [12]. Diese Kaskade kann eine vorbestehende koronare Herzerkrankung aggravieren sowie eine Plaqueruptur auslösen, und in der Folge einen Myokardinfarkt.

Ein akuter Myokardinfarkt mit klassischen Hebungszeichen im EKG erfordert eine invasive Abklärung mit Katheterangiographie und Intervention. Bei fehlender EKG-Dynamik kann die nichtinvasive Bildgebung mittels kardialer Computertomographie hilfreich sein, um in einem ersten Schritt die Obstruktion der großen epikardialen Gefäße auszuschließen. Weiterführend, sofern es der Zustand des Patienten bzw. die klinische Logistik zulässt, würde in der Abklärung einer Troponin-T-Erhöhung eine kardiale Magnetresonanztomographie folgen. Falls bei fehlender Koronarobstruktion eine ischämische Nekrose oder ein postischämisches Narbenmuster vorliegt, würde damit die Diagnose MINOCA gesichert werden [13]. Die Unterscheidung MINOCA/Myokarditis hat im weiteren Verlauf einen Einfluss auf die Behandlung.

#### Perikarditis

**Figure 2:**
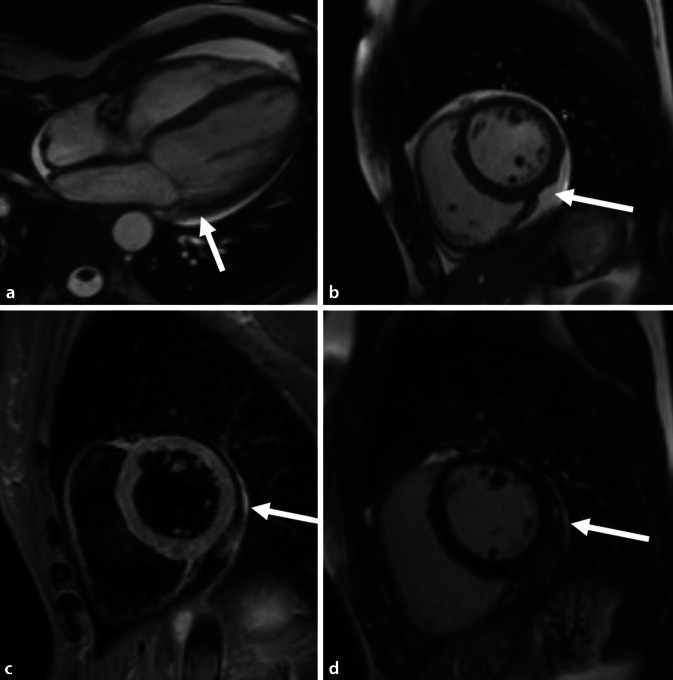

Neben der Affektion des Herzmuskels und der Gefäße wurde schon in initialen Fallserien eine Beteiligung des Perikards berichtet [10]. Perikardergüsse und perikardiale Kontrastmittelaufnahme wurden in bis zu 25 % der Patienten während oder nach einer COVID-19-Infektion beobachtet (14, Abb. 2). Bei pädiatrischen und adulten Patienten wurde dieser Prozentsatz mit 10–42 % beschrieben [15, 16].

Die Pathophysiologie der Perikarditis wird in erster Linie als Beteiligung im Rahmen einer Myokarditis gesehen. Bildgebend wird die Perikarditis anhand einer Perikardverdickung zusammen mit einer perikardialen Kontrastmittelaufnahme in der MRT nachgewiesen. Daneben kann auch die Durchführung einer Positronenemissionstomographie (PET) oder eine CT des Herzens hilfreich sein.

#### Arrhythmien

Arrhythmische Komplikationen im Rahmen einer COVID-19-Infektion werden in erster Linie durch die beschriebenen pathophysiologischen Vorgänge wie Fieber, Hypoxie, sympathische Aktivierung und Volumenbelastung getriggert. Sinustachykardien sind die am häufigsten beschriebenen Arrhythmien in Folge einer Infektion [17]. Daneben sind auch Bradykardien durch Schädigung des Reizleitungssystems und Vorhofflimmern in der Literatur beschrieben. Eine kardiale Arrhythmie wirkt sich zumeist auf die Auswurfleistung aus und verschlechtert somit die Hämodynamik, was wiederum Hypoxie und Volumenbelastung steigert.

Bei SARS-CoV-2-Patienten mit Arrhythmien können bildgebende Verfahren dazu dienen, nichtinvasiv Vorhof- oder Herzohrthromben auszuschließen oder den Grund der Arrhythmien zu bestimmen. Im Ausschluss von Vorhof- oder Herzohrthromben stellt die kardiale CT eine ideale Alternative zur transösophagealen Echokardiographie dar, da diese bei der Intubation mit einer potenziellen Aerosolbelastung für den Untersucher behaftet ist [8, 18].

Zur Darstellung der potenziellen Ursache einer Arrhythmie eignen sich sowohl die kardiale CT – zum Ausschluss einer obstruktiven koronaren Herzerkrankung – als auch die kardiale MRT – um eine Myokarditis oder eine postischämische Schädigung nachzuweisen.

### Bildgebung bei Long-COVID/Post-COVID

Neben dem Nachweis einer COVID-19-Beteiligung im Rahmen der akuten Phase wird es nun aktuell zunehmend wichtiger, postinflammatorische oder postischämische Organschäden nach durchgemachter Erkrankung nachzuweisen, um die Patienten in der Rehabilitationsphase nach ihrem Risiko entsprechend zu stratifizieren. Da sich nach COVID-19 pulmonale und kardiale Symptome oft vermischen, ist oft eine detaillierte Aufarbeitung mit Hilfe moderner Bildgebung erforderlich. Das Leitsymptom Dsypnoe könnte sowohl eine pulmonale (funktionell restriktiv, Restinfiltrate oder Narben), eine pulmonal vaskuläre (PE, Entwicklung einer CTEPH) als auch eine kardiale Ursache haben (verminderte Pumpleistung). Daneben hat der Nachweis einer postentzündlichen Myokardnarbe Konsequenzen für die Therapieplanung, vor allem bei Patienten mit Arhythmien nach durchgemachter Infektion. Eine initiale Abklärung erfolgt hier meist mittels Echokardiographie, zur genauen Myokardcharakterisierung bietet sich hier jedoch eher die kardiale MRT an. Neben der präzisen Funktionsanalyse insbesondere des rechten Ventrikels erlaubt sie einen genauen Nachweis von (Rest)Ödemzonen und Narben [8].

### Narbe nach Myokarditis

Die Darstellung einer Myokardnarbe nach Myokarditis mit LGE ist ein inzwischen etablierter Risikofaktor für ein verschlechtertes Outcome nach durchgemachter Erkrankung. Das Vorliegen einer Narbe erhöhte die Wahrscheinlichkeit klinischer Ereignisse wie Episoden einer kardialen Dekompensation bzw. Arrhythmien. Wie sich das Vorhandensein einer Narbe bei COVID-19-Patienten mit myokardialer Beteiligung auf ihre Prognose auswirkt, wird sich wohl erst in ein den nächsten Jahren erweisen. Langzeitanalysen aus Kohortenstudien mit Patienten nach durchgemachter, nicht COVID-19-assoziierter Myokarditis in Italien zeigten, dass das Vorhandensein einer postmyokarditischen Narbe, speziell im Bereich der anteroseptalen Wandabschnitte des linken Ventrikels (LV), mit einem schlechteren Outcome und einer höheren Rate an kardiovaskulären Ereignissen einhergeht [19].

Neben dem klassischen Nachweis einer Narbe mittels LGE wurden aber bereits auch weitere, neu etablierte kardiale Biomarker nach COVID-19 untersucht. Hierbei zeigten erste Analysen, dass Patienten auch nach relativ symptomfreier durchgemachter Infektion im Vergleich zu nicht COVID-19-Infizierten myokardial ein erhöhtes extrazelluläres Volumen zeigen [20]. Zudem zeigten diese Patienten einen reduzierten globalen longitudinalen Strain, der auf eine zunehmende Versteifung des Ventrikels hinweisen könnte. Ob diese Veränderungen prognostisch relevant sein könnten, werden Folgeuntersuchungen in den kommenden Jahren zeigen.

### Herzinsuffizienz

Neben der Darstellung von strukturellen Abnormitäten ist insbesondere die kardiale MRT aufgrund ihrer hohen Reproduzierbarkeit über die Zeit auch gut geeignet, um bei Patienten eine (beginnende) Herzinsuffizienz zu diagnostizieren und, wenn erforderlich, auch über die Zeit nachzuverfolgen. Zusätzlich zu den etablierten Laborparametern, wie z. B. dem „brain natiuretic peptid“ (BNP) bietet sich hier bildgebend neben den klassischen Funktionsparametern auch die Erfassung des myokardialen Strains als potenziellem Herzinsuffizienzbiomarker an.

Bei Patienten mit bereits vorbestehender Herzinsuffizienz verläuft eine SARS-CoV-2-Infektion meist schwerer und ist mit einer höheren Sterblichkeit assoziiert [3]. Zur Verschlechterung einer vorhandenen Herzinsuffizienz im Rahmen von COVID-19 tragen zahlreich Parameter bei. So wird der rechte Ventrikel über die Volumenbelastung und Druckerhöhung im Rahmen der pulmonalen Infektion beeinträchtigt. Der linke Ventrikel wiederum ist durch die genannten direkten Schädigungen im Rahmen einer Myokarditis belastet sowie durch ein erhöhtes Schlagvolumen im Rahmen einer Sepsis und in einem gewissen Grad durch Ischämie. Weitere Faktoren kommen hinzu, wie z. B. sympathische Aktivierung, systemische Inflammation und direkte Schädigung des Myokards durch applizierte Medikamente.

Die Bildgebung der Herzinsuffizienz im akuten Stadium einer Infektion basiert in erster Linie auf der Echokardiographie und wird mittels Schnittbildverfahren je nach Bedarf unterstützt werden [8]. In der postinfektiösen Phase bietet sich wiederum die kardiale MRT zur Detektion von strukturellen Abnormitäten an.

### Spezielle Kohorten nach SARS-CoV‑2-Infektionen

#### Leistungssportler

Leistungssportler stellen nach einer durchgemachten COVID-19-Infektion ein besonderes Patientengut dar. Hier ist es oft schwierig, eine pathologische von einer sportphysiologisch bedingten Vergrößerung des Herzens abzugrenzen, da zudem auch grenzwertig niedrige Auswurfwerte in Ruhe teilwiese als normal angesehen werden können. Nach durchgemachter SARS-CoV-2-Infektion könnte die zu frühe Wiederaufnahme von (Leistungs‑)Sport das Risiko für kardiale Ereignisse erhöhen. In einer initialen Publikation über nach COVID-19-genese Sportler wurde von einem hohen Prozentsatz von MR-Pathologien ausgegangen [21]. Eine initiale Serie an einer Kohorte von 26 Athleten zeigte auch einen relativ hohen Prozentsatz (15 %; *n* = 4) an Myokardpathologien, (bevorzugt Narben) in einer Kohorte von 26 Athleten. In darauffolgenden Serien mit jungen, eher symptomlosen Patienten konnte dies jedoch nicht bestätigt werden. Hier waren Pathologien mit 1,4–3 % sehr selten, sodass ein Screening mittels kardialer MRT in dieser Population derzeit nicht gerechtfertigt zu sein scheint [22, 23].

#### Pädiatrische Patienten nach COVID‑19

Bei Kindern verläuft eine SARS-CoV-2-Infektion oft mit eher milden pulmonalen oder gar ausschließlich gastrointestinalen Symptomen [16]. Dennoch kann es nach einigen Wochen zu einer überschießenden Immunreaktion kommen, die als „pediatric inflammatory multisystem syndrome“ (PIMS) bezeichnet wird. Dieses äußert sich klinisch als Konjunktivitis, Polyserositis und Gastroenteritis. Begleitend können kardiovaskuläre Symptome mit Hypotonie bis hin zu Schock, linksventrikulärer Dysfunktion sowie Perikardergüssen und Zeichen der Perikarditis auftreten. Bei den kardialen Manifestationen gibt es deutliche Parallelen zum Kawasaki-Syndrom, einer immungetriggerten Vaskulitis der mittelgroßen Arterien [24]. Im Rahmen des COVID-19-assoziierten PIMS wurden in ersten Fallserien mit Bildgebung ebenfalls kardiale Veränderungen nachgewiesen. Diese inkludierten temporäre linksventrikuläre Dysfunktion kombiniert mit einer Mitralinsuffizienz, Zeichen einer Perikarditis, Zeichen der Myokarditis bis hin zu kleinen Myokardinfarkten [16, 25]. Zusätzlich wurden Koronararterienektasien wie beim Kawasaki-Syndrom beschrieben, sogar kleine Infarkte [16].

### Myokarditis nach Impfung mit mRNA-Vakzinen

Zum Zeitpunkt des Verfassens des Artikels gab es mehrere Berichte von Fällen (v. a. in den USA und Israel) von Myokarditis und Perikarditis in Zusammenhang mit einer COVID-19-Impfung mit mRNA-basierten Impfstoffen (mRNA: Messenger-Ribonukleinsäure); [26]. Die Pathophysiologie der impfassoziierten Myokarditis ist noch unklar und scheint selten zu sein. Es sind vor allem junge, männliche Geimpfte betroffen, der Verlauf ist meist unkompliziert, und die Symptome klingen entweder spontan oder nach Behandlung mit Kortison rasch und vollständig ab [27]. Bildgebung und Diagnosekriterien dieser Form der Myokarditis unterscheiden sich nicht von einer direkt virusassoziierten Form und erfolgen nach den modifizierten Lake-Louise-Kriterien [7].

## Fazit für die Praxis

- Die kardiale Beteiligung einer COVID-19(Coronavirus-Krankheit 2019)-Infektion ist insbesondere bei Troponin-T-positiver mittelschwerer bis schwerer Erkrankung mit bis zu 50–60 % überaus häufig.
- Die kardiale Bildgebung mittels Computertomographie (CT) und Magnetresonanztomographie (MRT) des Herzens dient in der akuten Phase als wichtiges diagnostisches Werkzeug und wird zudem in der nahen Zukunft auch prognostisch relevante Information liefern.
- Symptome wie V. a. kardialen Thoraxschmerz, Arrhythmien bzw. Troponin-T-Auslenkungen im Labor sollten bei COVID-19 mittels kardialer Bildgebung weiter abgeklärt werden.
- Als wichtige kardiale COVID-19-Manifestationen sind Myokarditis, Perikarditis, akute Ischämie, Arrhythmien und die Entwicklung einer Herzinsuffizienz zu nennen.
- In der Aufarbeitung einer stattgehabten myokardialen Beteiligung bei Long-COVID-Patient(inn)en könnte der kardialen MRT eine zentrale Rolle zukommen.
- Diese stellt zudem eine wichtige Modalität zur Abklärung der impfassoziierten Myokarditis dar.
